# Supplementary material for: The interactome of intact mitochondria by cross-linking mass spectrometry provides evidence for coexisting respiratory supercomplexes
Source: Mol Cell Proteomics. 2017 Dec 8;17(2):216–32. doi: 10.1074/mcp.RA117.000470 (PMC5795388; doi:10.1074/mcp.RA117.000470)
Supplement: Supplemental Data [file supp_RA117.000470_133922_0_supp_23978_fzffwf.zip › spectra_annotation/mito_DR_spectra_annotation/175-1-3-1-32-1.pdf]

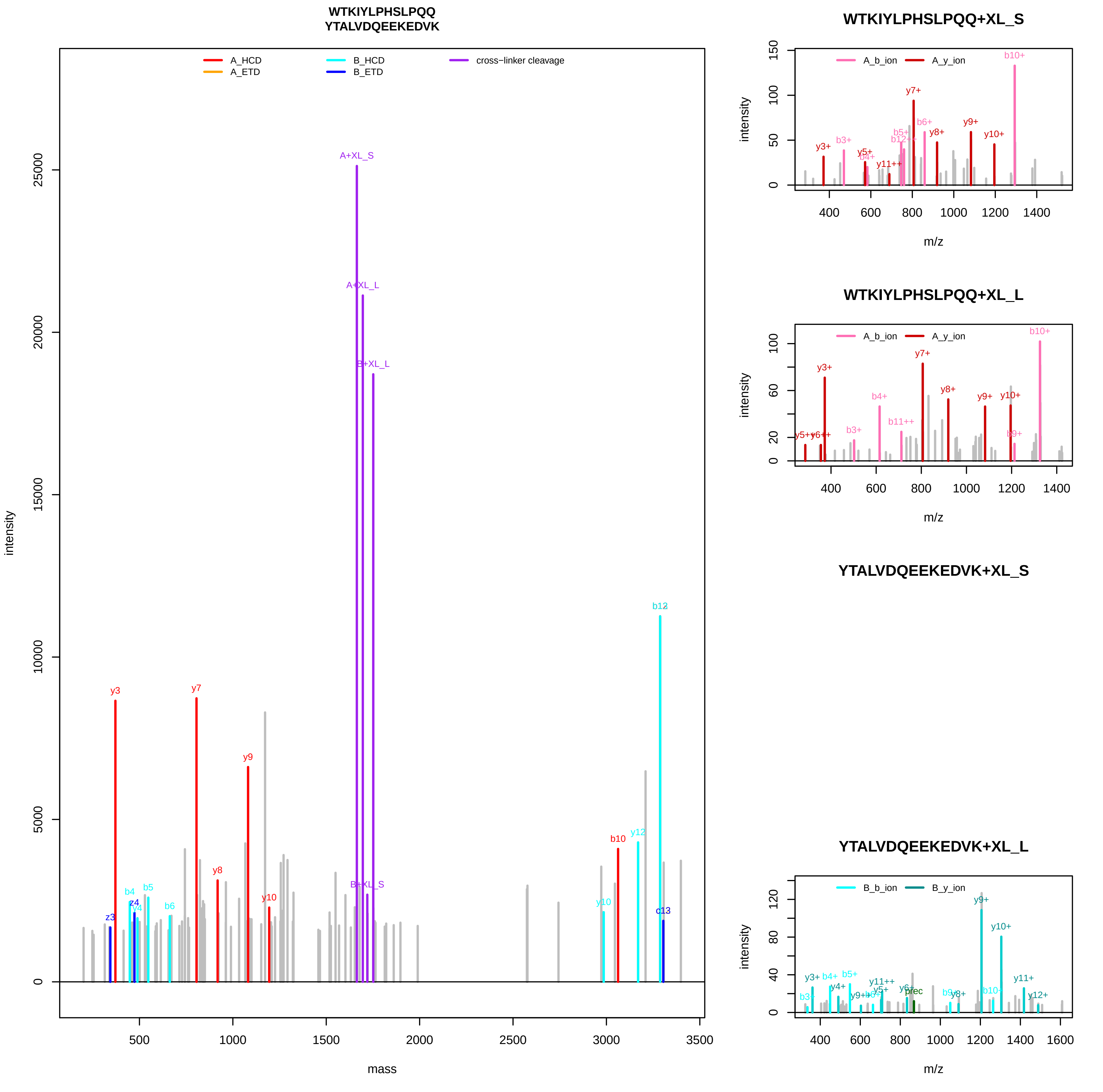
WTKIYLPHSLPQQ
YTALVDQEEKEDVK
A_HCD
A_ETD
B_HCD
B_ETD
cross−linker cleavage
A+XL_S
A+XL_L
B+XL_L
B+XL_S
y3
y7
y9
y8
y10
b10
b4
z4
b5
y4
z3
b6
y10
y12
b12
c13
intensity
mass
0
5000
10000
15000
20000
25000
500
1000
1500
2000
2500
3000
3500
WTKIYLPHSLPQQ+XL_S
A_b_ion
A_y_ion
b10+
y7+
b6+
y8+
y9+
y10+
b5+
b12++
y3+
b3+
y5+
b4+
y11++
m/z
400
600
800
1000
1200
1400
0
50
100
150
WTKIYLPHSLPQQ+XL_L
A_b_ion
A_y_ion
b10+
y7+
y3+
y8+
b4+
y9+
y10+
b11++
b3+
y5++
y6++
b9+
0
20
60
100
YTALVDQEEKEDVK+XL_S
YTALVDQEEKEDVK+XL_L
B_b_ion
B_y_ion
y9+
y10+
y3+
b4+
b5+
y4+
y11++
y9++
y5+
y6+
prec
b9+
y8+
b10+
y11+
y12+
b3+
0
40
80
120
1600
